
JOURNAL INFORMATION
==============================
NLM Title Abbreviation: Clin Infect Dis
Journal ID: Clin. Infect. Dis
NLM Journal ID: 9203213
Journal ID: cid

Clinical Infectious Diseases: An Official Publication of the Infectious Diseases Society of America

ISSN: 1058-4838
EISSN: 1537-6591

ARTICLE INFORMATION
==============================
PMCID: PMC7314248
PMID: 32533824
DOI: 10.1093/cid/ciaa747
Article ID: ciaa747
Article version: 1
Subjects: Editorial Commentary, AcademicSubjects/MED00290

Tuberculosis and type 2 Diabetes Mellitus: an inflammatory danger signal in the time of COVID-19

Wilkinson Robert J 1 2 3
1 The Francis Crick institute London , United Kingdom
2 Department of Infectious Diseases, Imperial College London , United Kingdom
3 Wellcome Center for Infectious Diseases Research in Africa, Institute of Infectious Disease and Molecular Medicine and Department of Medicine, University of Cape Town, Observatory, Republic of South Africa
Corresponding author: Robert.Wilkinson@Crick.ac.uk, +44 203 796 2306
Electronic publication date: 2020 Jun 13
Electronic Location ID: ciaa747
Received 2020 Jun 2
Copyright: © The Author(s) 2020. Published by Oxford University Press for the Infectious Diseases Society of America.
Copyright year: 2020
License: This is an Open Access article distributed under the terms of the Creative Commons Attribution License (http://creativecommons.org/licenses/by/4.0/), which permits unrestricted reuse, distribution, and reproduction in any medium, provided the original work is properly cited.
License URL: https://creativecommons.org/licenses/by/4.0/

Comment in: Impact of Intermediate Hyperglycemia and Diabetes on Immune Dysfunction in Tuberculosis 72 1 13 6 2020 69 78 Clinical Infectious Diseases: An Official Publication of the Infectious Diseases Society of America 10.1093/cid/ciaa751 PMC7823074 32533832
Comment in: Impact of Intermediate Hyperglycemia and Diabetes on Immune Dysfunction in Tuberculosis 72 1 13 6 2020 69 78 Clinical Infectious Diseases: An Official Publication of the Infectious Diseases Society of America 10.1093/cid/ciaa751 PMC7823074 32533832
article-lifecycle: PAP
edited-state: accepted-manuscript

==============================
### Tuberculosis and type 2 Diabetes Mellitus: an inflammatory danger signal in the time of COVID-19

Robert J. Wilkinson[1,2,3]

1. The Francis Crick institute London NW1 1AT, United Kingdom

2. Department of Infectious Diseases, Imperial College London W12 0NN, United Kingdom

3. Wellcome Center for Infectious Diseases Research in Africa, Institute of Infectious Disease and Molecular Medicine and Department of Medicine, University of Cape Town, Observatory 7925, Republic of South Africa

Robert.Wilkinson@Crick.ac.uk +44 203 796 2306

Accepted Manuscript

© The Author(s) 2020. Published by Oxford University Press for the Infectious Diseases Society of America.
This is an Open Access article distributed under the terms of the Creative Commons Attribution License (http://creativecommons.org/licenses/by/4.0/), which permits unrestricted reuse, distribution, and reproduction in any medium, provided the original work is properly cited.

There is association between type 2 Diabetes Mellitus (DM) and tuberculosis [1]. The association is becoming more significant due to the epidemiological transition as DM is growing rapidly in settings where tuberculosis is common. DM increases the risk of developing tuberculosis and is also associated with severe cavitating disease and adverse treatment outcomes, including death [2-5]. Tuberculosis increases insulin resistance and stress-induced hyperglycemia that may revert to normal during treatment [6, 7]. Testing for DM in tuberculosis patients is thus recommended, with confirmatory tests after 2–3 months of tuberculosis treatment initiation [8].

The risk of diabetics to infection has long been attributed to the hyperglycemic environment that favors immune dysfunction (e.g., impaired neutrophil function, depression of the antioxidant system, and humoral immunity), micro- and macro-angiopathies, neuropathy, decrease in the antibacterial activity of urine, gastrointestinal and urinary dysmotility, and greater number of medical interventions in such patients [9]. However it is increasingly appreciated DM itself is an inflammatory disorder that may therefore interact with infection in more complex ways [10].

Transcriptomic profiling of whole blood is a technique that has been widely applied and proven insightful in many infectious and inflammatory disorders [11]. Active tuberculosis has a transcriptomic signature dominated by a neutrophil-driven type 1 and 2 interferon-inducible gene profile. The transcriptomic signature relates to disease extent and resolves during successful treatment [12]. The differentiation of active from latent tuberculosis and other conditions may be aided by transcriptomic profiling [13]. The exaggerated inflammation that characterizes HIV-tuberculosis-associated immune reconstitution inflammatory syndrome is triggered by Toll-like receptor and inflammasome signalling [14]. Transcriptomic signatures may predict progression of, or detect, subclinical tuberculosis [15-17].

In this issue of the journal Eckold and colleagues present an analysis of the effect of DM and intermediate hyperglycaemia on the tuberculosis whole blood transcriptomic signature [INSERT REF.]. Samples were collected from active tuberculosis patients with DM (HbA1c ≥6.5%) or intermediate hyperglycaemia (IH, HbA1c 5.7-6.5%), tuberculosis-only

Accepted Manuscript patients and healthy controls in four countries: South Africa, Romania, Indonesia and Peru. Differential blood gene expression in 249 participants was determined by RNA sequencing. DM increased the magnitude of gene expression change in the host transcriptome in tuberculosis, notably showing an increase in genes associated with innate inflammatory and decrease in adaptive immune responses. Patients with intermediate hyperglycaemia and tuberculosis exhibited blood transcriptomes more similar to DM-tuberculosis patients than to patients with only tuberculosis. Both DM-tuberculosis and intermediate hyperglycaemia-tuberculosis patients had a decreased type I interferon response and a relatively stronger neutrophil component relative to tuberculosis-only patients, suggesting skewing of the TB transcriptome phenotype. Such immunological dysfunction is also present in individuals with intermediate hyperglycaemia, showing that altered immunity to tuberculosis may also be present in this group. The authors conclude that tuberculosis disease outcomes in individuals with IH diagnosed with TB should be investigated further.

The study has the advantage of power and of validation across populations. Limitations are that $HbA1_c$ is a relatively insensitive test for DM. It is known that tuberculosis itself associates with transiently impaired glucose tolerance which may even be in the frank diabetic range, yet which may resolve during tuberculosis treatment [7], but the proportion of cases of IH falling into this category was not investigated longitudinally.  Sex influences type 1 interferon and neutrophil transcript abundance and there were some very substantial gender differences between groups ranging from 18-90% males.

This publication coincides with the world grappling with the COVID-19 pandemic. Are there inferences that can be made from this very detailed study of susceptibility to tuberculosis conferred by diabetes? Male sex and diabetes have been identified in virtually every study as risk factors for severe COVID-19 infection, associated in the largest study to date with an adjusted hazard ratio for in-hospital death of 1.99 (male sex) and 1.50 for controlled (HbA1c < 58 mmol/mol) and 2.36 for uncontrolled DM [18]. The pulmonary innate immune response in COVID-19 patients with severe disease implicates a macrophage subpopulation with an interferon-associated hyperinflammatory response analogous to that observed in TB patients [19]. Type 1 and 2 interferon stimulation increases angiotensin converting enzyme 2 (ACE2), the receptor for SARS-CoV2, expression on respiratory epithelial cells. In particular, lungs of patients with HIV-associated TB show

Accepted Manuscript the highest expression of ACE2 in epithelial cells, compared to lungs of patients with TB only and healthy lungs [20]. HIV-1 infection, prior to antiretroviral therapy (ART) also induces a similar interferon signalling gene (ISG) profile to that of TB patients, a pattern also implicated in the context of HIV-TB co-infection, even prior to TB symptom onset [16]. Given these findings, it is plausible that individuals with HIV-1 and/or TB infection could be at increased risk of SARS-CoV2 infection not only via immunosuppression but also via increased ACE2 expression, predisposing to enhanced viral uptake. This theory is supported by a small observational study of 46 COVID-19 patients in China, which found active and latent TB infection (LTBI) predisposed to more severe presentations of COVID-19 as well as faster progression to clinical severity, and that TB infection was more common in COVID-19 pneumonia cases than that of other cases of bacterial and viral pneumonia within the same hospital [21]. Another recent report documents the coincidence of COVID-19 and tuberculosis [22].

Another aspect suggested by the pneumonia elicited by SARS-CoV2 infection is the capacity to enhance TB progression in subclinical and latently infected individuals.  The SARS-CoV2 immunological milieu, dominated by IL-1β, IL-6 and tumor necrosis factor (TNF), is determined by single cell RNA sequencing (to be orchestrated by inflammatory monocytes that differentiate into various macrophage sublineages, with the proportional representation of sublineages defining the difference between patients with mild or severe disease development [19]. Bronchoalveolar lavage from patients who developed severe pneumonia possessed a unique macrophage population that expressed high levels of inflammatory chemokines (CCL2, CCL3, CCL4, CXCL9, CXCL10, CXCL11) and ISGs (APOBEC3A, ISG15, ISG20, GBP1, GBP5, IFITM3, MX1) which are similarly upregulated in TB and HIV-TB patients [16]. Thus, the lung immunological milieu elicited in COVID-19 patients has the potential to exacerbate TB immunopathogenesis in those with latent TB infection, and increase their risk of TB progression.

Lastly neutrophil activation in the form of release of neutrophil extracellular traps (NET) has also been suggested to contribute to the severe pneumonia of COVID-19. Neutrophilia also predicts poor outcome in patients with COVID-19. In autopsy samples from 3 COVID-19 patients, neutrophil infiltration into pulmonary capillaries with fibrin deposition and extravasation into the alveolar space was observed [23]. In addition the sera of patients with COVID-19 patients have elevated levels of myeloperoxidase-DNA, and citrullinated

Accepted Manuscript histone H3; both specific markers of NET formation [24]. Thus the factors that underly neutrophilic skewing of the transcriptomic response of diabetics to tuberculosis may also contribute to severe pathology in COVID-19.

What are the practical consequences? Lower- and middle-income countries are as yet to experience the full brunt of the COVID-19 epidemic. Yet many already have the rising coincidence of DM and endemic tuberculosis. Routine services for both diseases have been disrupted by the initial necessarily vertical approach to COVID-19 control. Good glycaemic control should be emphasised in known diabetics as well as stringent social distancing and shielding as necessary. There is no global policy on the treatment of latent tuberculosis in diabetics: a priority question now urgently in need of evidence. Longitudinal clinico-epidemiologic studies or registers of overlap between DM, COVID-19 and tuberculosis are pressing to advise risk and potential need to change policy.

Accepted Manuscript

Acknowledgements

RJW is supported by the Francis Crick Institute which receives funding from Wellcome (FC0010218), UKRI (FC0010218) and CRUK (FC0010218). He also receives support from Wellcome (014803 and 203135), NIH (R01AI145436) and EDCTP (RIA2017T-2004). The views in this article are the author's alone and do not reflect the views of these agencies.

Accepted Manuscript

**References**

1. Jeon CY, Murray MB. Diabetes mellitus increases the risk of active tuberculosis: a systematic review of 13 observational studies. PLoS Med **2008**; 5(7): e152.
2. Shewade HD, Jeyashree K, Mahajan P, et al. Effect of glycemic control and type of diabetes treatment on unsuccessful TB treatment outcomes among people with TB-Diabetes: A systematic review. PLoS One **2017**; 12(10): e0186697.
3. Baker MA, Harries AD, Jeon CY, et al. The impact of diabetes on tuberculosis treatment outcomes: a systematic review. BMC Med **2011**; 9: 81.
4. Faurholt-Jepsen D, Range N, PrayGod G, et al. Diabetes is a strong predictor of mortality during tuberculosis treatment: a prospective cohort study among tuberculosis patients from Mwanza, Tanzania. Trop Med Int Health **2013**; 18(7): 822-9.
5. Moreira J, Castro R, Lamas C, Ribeiro S, Grinsztejn B, Veloso VG. Hyperglycemia during tuberculosis treatment increases morbidity and mortality in a contemporary cohort of HIV-infected patients in Rio de Janeiro, Brazil. Int J Infect Dis **2018**; 69: 11-9.
6. Dungan KM, Braithwaite SS, Preiser JC. Stress hyperglycaemia. Lancet **2009**; 373(9677): 1798-807.
7. Kubjane M, Berkowitz N, Goliath R, Levitt NS, Wilkinson RJ, Oni T. Tuberculosis, HIV and the association with transient hyperglycaemia in peri-urban South Africa. Clin Infect Dis **2019**.
8. Ottmani SE, Murray MB, Jeon CY, et al. Consultation meeting on tuberculosis and diabetes mellitus: meeting summary and recommendations. Int J Tuberc Lung Dis **2010**; 14(12): 1513-7.
9. Casqueiro J, Casqueiro J, Alves C. Infections in patients with diabetes mellitus: A review of pathogenesis. Indian J Endocrinol Metab **2012**; 16 Suppl 1: S27-36.
10. Hodgson K, Morris J, Bridson T, Govan B, Rush C, Ketheesan N. Immunological mechanisms contributing to the double burden of diabetes and intracellular bacterial infections. Immunology **2015**; 144(2): 171-85.

Accepted Manuscript

11. Singhania A, Wilkinson RJ, Rodrigue M, Haldar P, O'Garra A. The value of transcriptomics in advancing knowledge of the immune response and diagnosis in tuberculosis. Nat Immunol **2018**; 19(11): 1159-68.

12. Berry MP, Graham CM, McNab FW, et al. An interferon-inducible neutrophil-driven blood transcriptional signature in human tuberculosis. Nature **2010**; 466(7309): 973-7.

13. Kaforou M, Wright VJ, Oni T, et al. Detection of tuberculosis in HIV-infected and -uninfected African adults using whole blood RNA expression signatures: a case-control study. PLoS Med **2013**; 10(10): e1001538.

14. Lai RP, Meintjes G, Wilkinson KA, et al. HIV-tuberculosis-associated immune reconstitution inflammatory syndrome is characterized by Toll-like receptor and inflammasome signalling. Nat Commun **2015**; 6: 8451.

15. Esmail H, Lai RP, Lesosky M, et al. Characterization of progressive HIV-associated tuberculosis using 2-deoxy-2-[18F]fluoro-D-glucose positron emission and computed tomography. Nat Med **2016**; 22(10): 1090-3.

16. Esmail H, Lai RP, Lesosky M, et al. Complement pathway gene activation and rising circulating immune complexes characterize early disease in HIV-associated tuberculosis. Proc Natl Acad Sci U S A **2018**; 115(5): E964-E73.

17. Singhania A, Verma R, Graham CM, et al. A modular transcriptional signature identifies phenotypic heterogeneity of human tuberculosis infection. Nat Commun **2018**; 9(1): 2308.

18. Williamson E, Walker AJ, Bhaskaran KJ, et al. OpenSAFELY: factors associated with COVID-19-related hospital death in the linked electronic health records of 17 million adult NHS patients. medRxiv **2020**: 2020.05.06.20092999.

19. Liao M, Liu Y, Yuan J, et al. Single-cell landscape of bronchoalveolar immune cells in patients with COVID-19. Nat Med **2020**.

20. Ziegler CGK AS, Nyquist SK, Mbano I, et al. SARS-CoV-2 receptor ACE2 is an interferon-stimulated gene in human airway epithelial cells and is enriched in specific cell subsets across tissues. Cell, under review, https://papers.ssrn.com/sol3/papers.cfm?abstract_id=3555145.

Accepted Manuscript

21. Liu Y BL, Chen Y, Wang Y, et al. Active or latent tuberculosis increases susceptibility to COVID-19 and disease severity. MedRxiv preprint doi: https://doi.org/10.1101/2020.03.10.20033795.

22. Tadolini M, Codecasa LR, García-García JM, et al. Active tuberculosis, sequelae and COVID-19 co-infection: first cohort of 49 cases. Eur Respir J **2020**.

23. Barnes BJ, Adrover JM, Baxter-Stoltzfus A, et al. Targeting potential drivers of COVID-19: Neutrophil extracellular traps. J Exp Med **2020**; 217(6).

24. Zuo Y, Yalavarthi S, Shi H, et al. Neutrophil extracellular traps in COVID-19. JCI Insight **2020**.

Accepted Manuscript
